# Chronic expanding hematoma in the retroperitoneal space: a case report

**DOI:** 10.1186/1471-2490-13-60

**Published:** 2013-11-18

**Authors:** Takahiro Syuto, Motoaki Hatori, Nomura Masashi, Yoshitaka Sekine, Kazuhiro Suzuki

**Affiliations:** 1 Department of Urology, Gunma University Graduate School of Medicine, Maebashi, Gunma 371-8511, Japan

**Keywords:** Hematoma, Retroperitoneal, Hydronephrosis

## Abstract

**Background:**

Chronic expanding hematoma is a rare condition that develops after surgery, trauma, or injury. It can also develop at any location in the body in the absence of trauma. Clinical findings and various diagnostic imaging modalities can aid in the differential diagnosis of this condition. In general, hematomas are naturally reabsorbed and rarely cause serious problems. However, hematomas that develop slowly without a history of trauma, surgery, or bleeding disorders could be difficult to differentiate from soft tissue neoplasms. In the present case, we describe a patient, without any history or physical evidence of trauma, who exhibited a large chronic expanding hematoma in the retroperitoneal space that resulted in hydronephrosis because of the pressure exerted on the left ureter.

**Case presentation:**

A 69-year-old man presented to our hospital with a swollen lesion in the left flank. A mass, 19 cm in diameter, was detected in the retroperitoneal space by computed tomography. We suspected the presence of a chronic expanding hematoma, soft tissue tumor, or left renal artery aneurysm. Surgical treatment was performed. However, postoperative histopathological examination indicated that the mass was a nonmalignant chronic expanding hematoma. No recurrence was observed during a 2-year follow-up period.

**Conclusion:**

In patients without a history of trauma who present slowly growing masses, the differential diagnosis should include chronic expanding hematoma in addition to cysts and soft tissue tumors. Moreover, the use of magnetic resonance imaging and computed tomography is essential to differentiate between chronic expanding hematoma and soft tissue tumors.

## Background

Hematomas can develop in many locations of the body as a result of trauma, surgery, or bleeding disorders. The diagnosis of this condition is based on medical history, physical findings, and the results of examinations involving various imaging modalities. Some hematomas persist as slowly expanding, space-occupying masses for months or years, and are termed as chronic expanding hematomas (CEH) [1]. In general, hematomas are naturally reabsorbed and rarely cause serious problems. However, those that develop slowly and progressively in patients with no history of trauma, surgery, or associated bleeding disorders can be difficult to differentiate from soft tissue neoplasms [2]. In the present report, we describe a case of CEH developing in the retroperitoneal space that was associated with hydronephrosis.

## Case presentation

A 69-year-old man with no prior history of medication or anticoagulant therapy first noticed a painless swelling in his left flank in 2005. This swelling gradually increased over 6 years, resulting in the formation of a mass in the left flank region. In June 2011, the patient presented to a local hospital after mild pain developed. The patient did not have any definite history of trauma or surgery that could have caused the left abdominal/back lesion. Computed tomography (CT) indicated the presence of a left retroperitoneal mass with left hydronephrosis because of external compression by the mass and a right atrophic kidney. A double J-stent was placed in the left ureter. The serum creatinine level changed from 0.72 mg/dL before double J-stent insertion to 0.53 mg/dL after the treatment.

**Figure 1:**
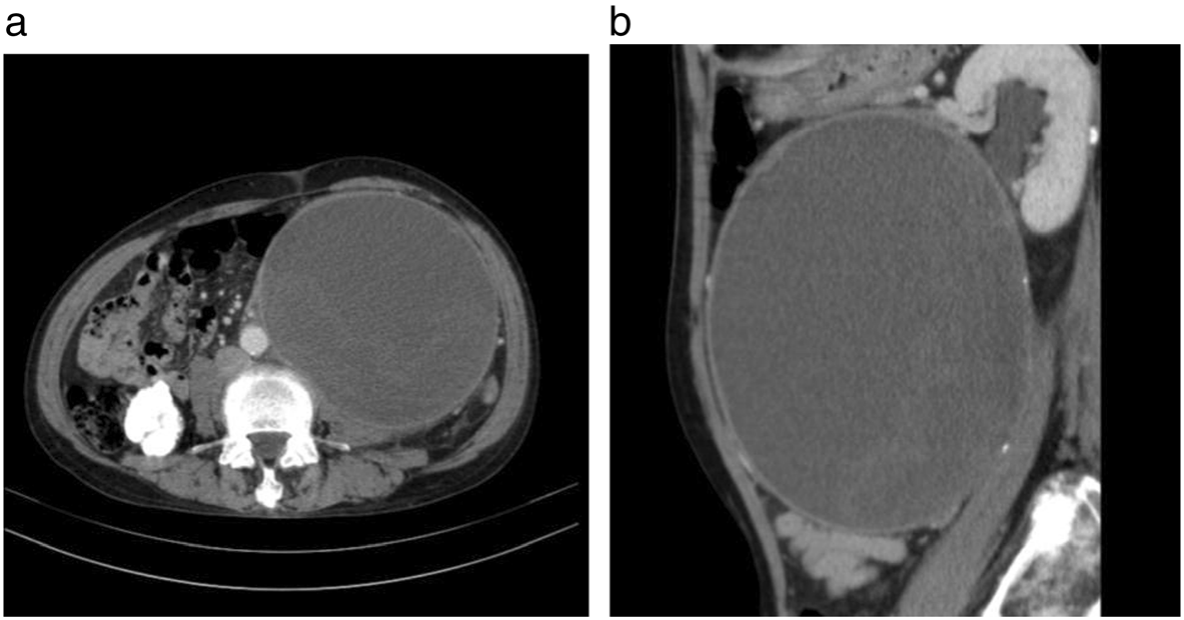
**Enhanced abdominal computed tomography. (a)** Axial view: The retroperitoneal mass is 20 cm × 15 cm × 13 cm in size and well-circumscribed. **(b)** Sagital view: The left kidney is compressed upward, and hydronephrosis is evident.

**Figure 2:**
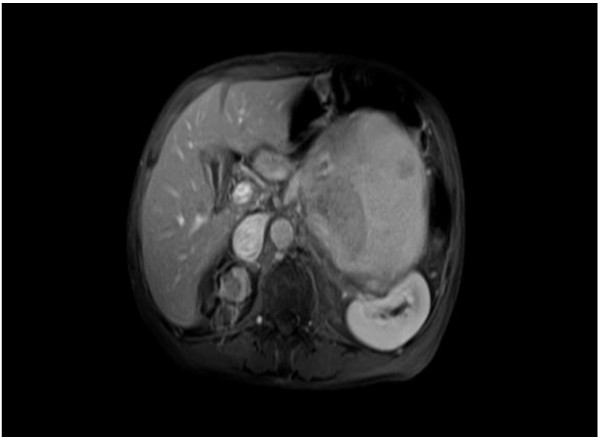
**Abdominal magnetic resonance imaging (MRI).** The T1-weighted images show high signal intensity and many partition walls.

**Figure 3:**
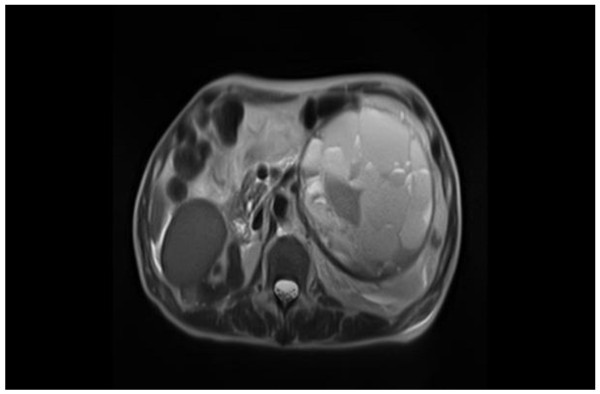
**Abdominal MRI.** The T2-weighted images show high signal intensity in a major portion of the mass. Heterogeneous signal intensity with hyperintense and hypointense areas is also observed.

On physical examination at the Gunma University Hospital, a firm area of swelling with an approximate size of 20 cm × 15 cm was detected. Blood tests results indicated that the patient had mild anemia and a hemoglobin level of 10.7 g/dL. The patient’s coagulation profile and platelet count were normal. However, CT indicated the presence of a huge mass (20 cm × 15 cm × 13 cm) compressing the left kidney in the upward direction in the retroperitoneal space. Contrast-enhanced CT revealed that the mass was not uniform but included scattered calcification and a partly enhanced rim (Figure 1). On T-1 and T-2 weighted magnetic resonance imaging (MRI), high signal intensity and several partition walls were observed in a major portion of the mass (Figures 2 and 3). No bone metastases were noted on bone scintigraphy.

**Figure 4:**
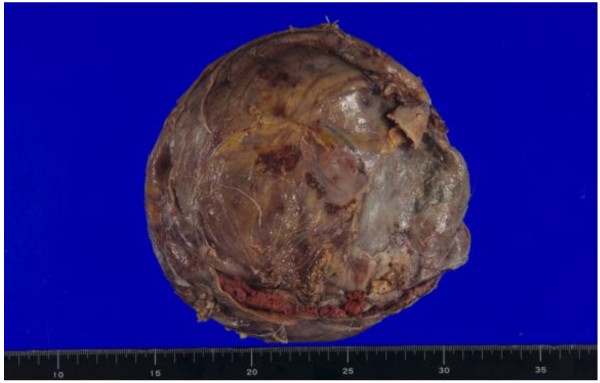
Gross lesion with a hard connective tissue capsule.

**Figure 5:**
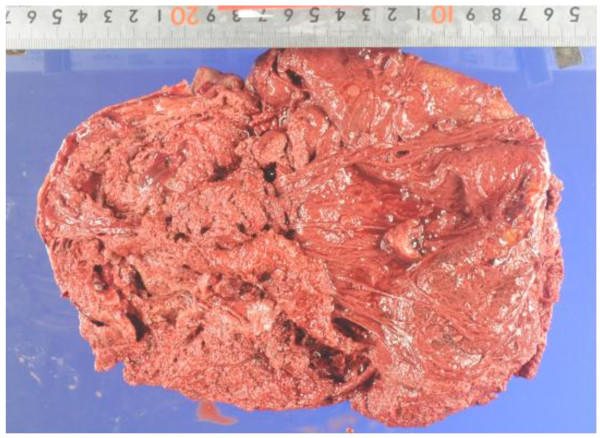
An old clot and friable material that was contained within the hematoma.

**Figure 6:**
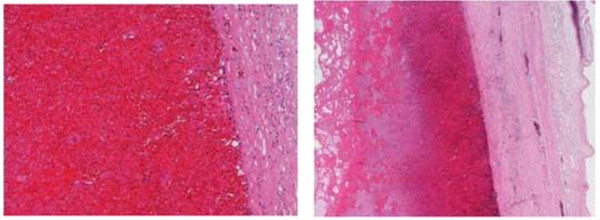
**Microscopic examination indicating the presence of fibroblastic granulation tissue and degenerative erythrocytes within the hematoma, which are surrounded by thick collagenous fibrous tissue.**

Based on these results and the patient’s clinical history, we suspected the presence of CEH, in addition to soft tissue malignancy and hemangiomas. Open abdominal surgery via the intraperitoneal approach was then performed. The mass was located in the retroperitoneal space, was completely encapsulated, and did not exhibit any evidence of invasion to the neighboring tissue; however, it was found to be partly adhering to the left ureter and psoas muscle. On exploration, we observed that there was no vascular malformation in the surrounding tissue. Although a slight adhesion was present between the mass and the left ureter, dissection was performed relatively easily because of a ureteral stent that was placed prior to the operation. However, the mass was partially adhering to the aorta, and therefore, we decided to perform dissection at a site close to the mass. Despite this careful dissection, the traction exerted on the thin capsule of the mass resulted in its rupture. We noted that the mass was filled with a dark brown substance including blood cells, necrotic tissue, and fibrin (Figures 4 and 5). Histopathological examination of the mass capsule revealed dense fibrous connective and fatty tissue containing numerous old clots (Figure 6). No evidence of neoplasia was found. The postsurgical diagnosis was a CEH in the retroperitoneal space. During the postoperative period, the patient developed high fever and a subcutaneous emphysema that spread out from the left thoracic wall to the left abdominal wall. As a result of consulting a dermatologist, a subcutaneous emphysema seemed to be caused by the operative stress than a bacterial infection. He received compression therapy by a compression garment. The laboratory data revealed leukocytosis and elevated levels of β-D-glucan. Antibiotic therapy with cefazolin was replaced with doripenem and clindamycin. In addition, treatment with fosfluconazole was initiated. We could not locate the site of inflammation or infection after examination by CT, urine culture, sputum culture, and blood culture. Thereafter, the fever and subcutaneous emphysema resolved, and the β-D-glucan levels gradually declined until normalization. No sign of CEH recurrence was observed 2 years after surgery.

## Discussion

CEH is a type of hematoma that is most commonly caused by trauma, and has certain other etiologies such as hemorrhagic disorder. Hematomas are often reabsorbed, and gradually decrease in size. However, in rare cases, they may develop slowly and expand progressively over a period of time. In certain cases, CEH may persist and increase in size for more than 1 month after the initial hemorrhagic event [1]. Hematomas in the skeletal muscles or surrounding tissue may develop as a result of a direct shearing force that splits the subcutaneous fat from the underlying fascia, thus potentially creating a large space, which may then fill with blood. Labadie et al. [3] reported that blood and erythrocyte degradation products, hemoglobin, leukocytes, platelets, and fibrin exert an irritant effect on the surrounding tissue. These factors are believed to induce a mild inflammatory response, which increases vascular wall permeability and bleeding from dilated capillaries in the granulation tissue beneath the capsular wall, thus resulting in the subsequent growth of the hematoma. However, no trigger such as trauma or anticoagulant therapy was identified in the present case.

Careful examination of a PubMed database search of articles published from 1970 to 2012 using the key words “chronic expanding hematoma” yielded 204 cases of CEH. Of these 204 cases, 79 cases were detected in the brain and spine; 59 were detected in the thorax; 56 were detected in subcutaneous tissues and muscles of the arms and legs; and 10 were detected in the abdomen, of which 7 were located in the retroperitoneal space (Table 1) [1,2,4-7]. None of the cases listed above presented with hydronephrosis. In the present case, the unusual size of the retroperitoneal lesion may have resulted in compression of the left ureter and kidney.

**Table 1:** Reported cases of retropritoneal chronic expanding hematomas

**Author**	**Patient age (years)**	**Patient sex**	**Site of the hematoma**	**Hematoma size (cm)**
Kaneko et al [4]	34	F	Above the right kidney	12
Hamada et al [5]	65	M	At the right iliac fossa	8
Irisawa et al [7]	70	M	Below the right kidney	18
Yamazaki et al [2]	53	M	Above the left kidney	12
Yamada et al [6]	59	M	Above the left kidney	12
Reid et al [1]	79	M	At the right iliac fossa	NA
Reid et al [1]	NA	NA	NA	8
Syuto et sl	69	M	Below the left kidney	20

*M*, male; *F*, female; *NA*, data not available.

CEH may be difficult to differentiate from soft tissue tumors (such as hemangiopericytomas and cavernous hemangiomas), sarcomas, actinomycosis, and inflammatory pseudotumors [7]. Weiss et al. [8] reported that hematomas are associated with approximately 5% of malignant fibrous histiocytomas.

Various imaging modalities have been used for the diagnosis of CEH. It has been stated that a dynamic CT scanning can detect a rim enhancement in the arterial phase in such cases, because granulation tissue with vascular channels is distributed within the hematoma capsule [2,9]. In the present case, enhanced CT revealed a partly enhanced rim. Although MRI is inferior to CT in identifying calcification or spatial resolution, MRI is more sensitive than CT in the diagnosis of hematomas. The signal within the lesion on MRI can vary with the passage of time, indicating time-related changes in hemoglobin levels. High signal intensity on T1-weighted images are attributable to the presence of methemoglobin within the hematoma. A few soft tissue tumors such as lipomas, liposarcomas, and hemangiomas also yield enhanced high signal intensity on T1-weighted images. However, it can be difficult to differentiate hematomas from malignant soft tissue tumors based on clinical and radiological findings because of the time-related changes in MRI signals [10]. Liu et al. reported that CEH should be considered in the differential diagnosis for soft tissue masses that exhibit internal hemorrhage and fibrous pseudocapsule during unenhanced T1- and T2-weighted MRI. If the contrast enhancement is patchy within the lesion, a diagnosis of hemorrhagic sarcomas should be considered [11]. In the present case, high signal intensity was predominantly observed on both T1-and T2-weighted images, except for an area of low signal intensity that represented a wall of collagenous fibrous tissue on the peripheral rim. A T2-weighted image of MRI in the present case showed a “mosaic sign,” which meant that the lesion involved repeated bleeding because it contained a mosaic of various signal intensities representing fresh and old blood [12]. These atypical MRI findings indicated the presence of CEH.

The optimal treatment option for CEH is complete excision of the hematoma together with its fibrous capsule. Aspiration of the liquid or drainage could result in serious bleeding or recurrence [9,13]. However, hematomas are often difficult to remove because of adhesion to the surrounding tissue and abundant neovascularization beneath the capsule. By using CT in particular, the presence of new capillaries and granulation tissue can be easily identified if contrast material is used [9].

In the present case, the left kidney was hydronephrotic. Therefore, a double J-stent was placed in the left ureter to aid in identifying and preventing injury to the left ureter. The double J-stent was removed 2 months postoperatively, and the hydronephrosis in the left kidney had resolved. To our knowledge, a hematoma of a comparable size as the one reported in the present case, accompanied with left hydronephrosis, has never been reported in the literature. Moreover, in the present case, the etiology of CEH was unclear and could not be determined during surgery. Although no recurrence was evident at the 2-year follow-up, it is essential to further follow-up the patient carefully.

## Conclusion

We reported the case of a patient with a huge CEH located in the retroperitoneal space that resulted in hydronephrosis. MRI may be useful for differentiating between CEH and malignant soft tissue tumors. Complete removal of the CEH resulted in resolution of hydronephrosis and no recurrence of CEH at the 2-year follow-up.

### Consent

Written informed consent was obtained from the patient for publication of this Case report and any accompanying images.

## Abbreviations

CEH: Chronic expanding hematomas; CT: Computed tomography; MRI: Magnetic resonance imaging.

## Competing interests

The authors declare that they have no competing interests.

## Authors’ contributions

TK was responsible for acquisition of data, drafting of manuscript and preparation of the figures and table. MH and MN operated the patient and revised manuscript. TS and KS was responsible for critical revision of the manuscript. All authors read and approved the final manuscript.

## Pre-publication history

The pre-publication history for this paper can be accessed here:

http://www.biomedcentral.com/1471-2490/13/60/prepub
